# *N* ^6^-(4-Fluoro­benz­yl)-3-nitro­pyridine-2,6-diamine

**DOI:** 10.1107/S1600536811018642

**Published:** 2011-05-20

**Authors:** Ji-long Ge, Xiao-min Qian

**Affiliations:** a Changzhou Siyao Pharmaceuticals Co. Ltd, Changzhou 213004, People’s Republic of China; b Ordered Matter Science Research Center, College of Chemistry and Chemical Engineering, Southeast University, Nanjing 210096, People’s Republic of China

## Abstract

In the title compound, C_12_H_11_FN_4_O_2_, the pyridine ring is connected to a benzene ring by a –CH_2_—NH_2_- chain. The nitro group is twisted out of the pyridine ring plane [torsion angle O—N—C—C = 10.41 (10)°]. An intramolecular N—H⋯O hydrogen bond occurs. The fluoro­benzene ring is disordered over two positions [occupancy ratio = 0.59 (3):0.41 (3)]. Inter­molecular N—H⋯O and N—H⋯N hydrogen bonds stabilize the crystal structure.

## Related literature

The title compound is an inter­mediate in the synthesis of analgesic drugs. For the analgesic properties of flupirtine (systematic name eth­yl{2-amino-6-[(4-fluoro­benz­yl)amino]­pyridin-3-yl}carbamate), see: Klawe & Maschke (2009 ▶). For synthetic procedures, see: Gerhard & Ilia (2010 ▶). For a related structure, see: Wang (2009 ▶).
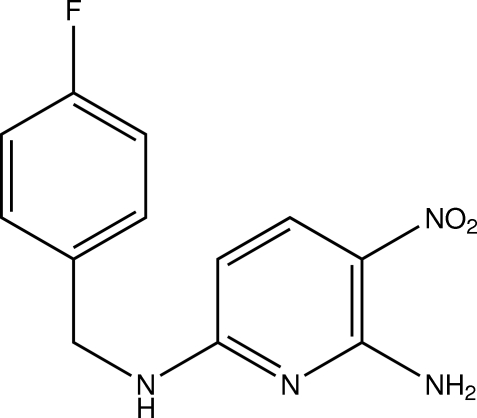


## Experimental

### 

#### Crystal data

C_12_H_11_FN_4_O_2_
*M* *_r_* = 262.25
Monoclinic, 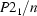

*a* = 14.8187 (14) Å
*b* = 5.9972 (6) Å
*c* = 14.8840 (15) Å
β = 109.827 (1)°
*V* = 1244.3 (2) Å^3^
*Z* = 4
Mo *K*α radiation
μ = 0.11 mm^−1^
*T* = 298 K
0.38 × 0.15 × 0.11 mm

#### Data collection

Rigaku SCXmini CCD diffractometer
Absorption correction: multi-scan (*CrystalClear*; Rigaku, 2005 ▶) *T*_min_ = 0.960, *T*_max_ = 0.988
5923 measured reflections
2184 independent reflections
1169 reflections with *I* > 2σ(*I*)
*R*_int_ = 0.056

#### Refinement

*R*[*F* ^2^ > 2σ(*F* ^2^)] = 0.052
*wR*(*F* ^2^) = 0.140
*S* = 1.03
2184 reflections
227 parameters
H-atom parameters constrained
Δρ_max_ = 0.21 e Å^−3^
Δρ_min_ = −0.22 e Å^−3^

### 

Data collection: *CrystalClear* (Rigaku, 2005 ▶); cell refinement: *CrystalClear*; data reduction: *CrystalClear*; program(s) used to solve structure: *SHELXS97* (Sheldrick, 2008 ▶); program(s) used to refine structure: *SHELXL97* (Sheldrick, 2008 ▶); molecular graphics: *ORTEP-3 for Windows* (Farrugia, 1997 ▶) and *PLATON* (Spek, 2009 ▶); software used to prepare material for publication: *SHELXL97*.

## Supplementary Material

Crystal structure: contains datablocks I, global. DOI: 10.1107/S1600536811018642/pv2411sup1.cif

Structure factors: contains datablocks I. DOI: 10.1107/S1600536811018642/pv2411Isup2.hkl

Supplementary material file. DOI: 10.1107/S1600536811018642/pv2411Isup3.cml

Additional supplementary materials:  crystallographic information; 3D view; checkCIF report

## Figures and Tables

**Table 1:** Hydrogen-bond geometry (Å, °)

*D*—H⋯*A*	*D*—H	H⋯*A*	*D*⋯*A*	*D*—H⋯*A*
N3—H3*B*⋯O1	0.86	2.03	2.651 (3)	129
N3—H3*A*⋯N1^i^	0.86	2.17	3.028 (3)	174
N2—H2⋯O1^ii^	0.86	2.35	3.060 (3)	141

Symmetry codes: (i) 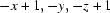; (ii) 
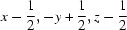
.

**Fig. 1.**
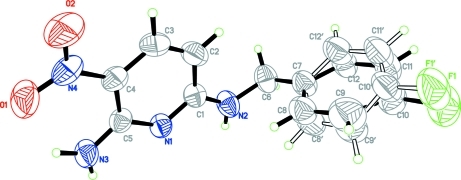
An ORTEP (Farrugia, 1997) view of the title compound with the atom-numbering scheme. Displacement ellipsoids are drawn at the 30% probability level. Smaller fraction of the fluorobenzene ring has been plotted with hollow bonds.

**Fig. 2.**
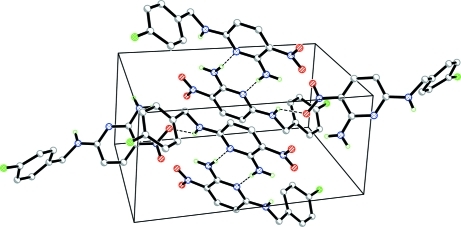
A packing diagram of the title compound showing hydrogen bonds. Smaller fraction of the fluorobenzene ring has been excluded.

## supplementary crystallographic information

### Comment

Flupirtine, ethyl{2-amino-6-[(4-fluorobenzyl)amino]pyridin-3-yl}carbamate, is of great importance owing to its analgesic properties (Klawe & Maschke, 2009). In this article, we report the crystal structure of the title compound which is one of the key intermediates in the synthsis of analgesia drugs (Gerhard & Ilia, 2010).

In the title molecule (Fig. 1), a pyridine ring is connected with a benzene ring by –CH_2_—NH_2_- chain and the nitro group is twisted out of the pyridine ring plane [torsion angle O1—N4—C4—C5 = 10.41 (10)°]. The crystal structure is stabilized by intermolecular N—H···O and N—H···N hydrogen bonds (Figure 2 and Table 1).

### Experimental

To a solution of 2-amino-3-nitro-6-chloropyridine (7.8 g, 45 mmol) in 2-propanol (50 ml) were added 4-fluorobenzylamine (5.63 g, 45 mmol) and triethylamine (6.45 g,64 mmol) (Gerhard & Ilia, 2010). Then another 30 ml 2-propanol was add to the above solution. The mixture was heated to backflow and stirred for 3 h. Then 100 ml water was add to the mixture to obtain the title compound which was recrystallized from ethanol by slow evaporation (yield 10.2 g, 91%).

### Refinement

H atoms bonded to C atoms were placed geometrically and treated as riding, with C—H = 0.93 (benzene ring) or 0.97 Å (methylene) and N—H = 0.86 Å with *U*_iso_(H) = 1.2*U*_eq_(C or N) or *U*_iso_(H) = 1.5*U*_eq_(methylene). The fluoro benzene ring was disordered over two positions with site occupancy factors 0.59 (3) and 0.41 (3).

### Crystal data

**Table:**

C_12_H_11_FN_4_O_2_	*F*(000) = 544
*M**_r_* = 262.25	*D*_x_ = 1.400 Mg m^−^^3^
Monoclinic, *P*2_1_/*n*	Mo *K*α radiation, λ = 0.71073 Å
Hall symbol: -P 2yn	Cell parameters from 1144 reflections
*a* = 14.8187 (14) Å	θ = 2.4–26.9°
*b* = 5.9972 (6) Å	µ = 0.11 mm^−^^1^
*c* = 14.8840 (15) Å	*T* = 298 K
β = 109.827 (1)°	Prism, yellow
*V* = 1244.3 (2) Å^3^	0.38 × 0.15 × 0.11 mm
*Z* = 4	

### Data collection

**Table:**

Rigaku SCXmini CCD diffractometer	2184 independent reflections
Radiation source: fine-focus sealed tube	1169 reflections with *I* > 2σ(*I*)
graphite	*R*_int_ = 0.056
Detector resolution: 8.192 pixels mm^-1^	θ_max_ = 25.0°, θ_min_ = 2.4°
φ and ω scans	*h* = −14→17
Absorption correction: multi-scan (*CrystalClear*; Rigaku, 2005)	*k* = −5→7
*T*_min_ = 0.960, *T*_max_ = 0.988	*l* = −17→17
5923 measured reflections	

### Refinement

**Table:**

Refinement on *F*^2^	Primary atom site location: structure-invariant direct methods
Least-squares matrix: full	Secondary atom site location: difference Fourier map
*R*[*F*^2^ > 2σ(*F*^2^)] = 0.052	Hydrogen site location: inferred from neighbouring sites
*wR*(*F*^2^) = 0.140	H-atom parameters constrained
*S* = 1.02	*w* = 1/[σ^2^(*F*_o_^2^) + (0.0585*P*)^2^] where *P* = (*F*_o_^2^ + 2*F*_c_^2^)/3
2184 reflections	(Δ/σ)_max_ = 0.001
227 parameters	Δρ_max_ = 0.21 e Å^−^^3^
0 restraints	Δρ_min_ = −0.22 e Å^−^^3^

### Special details

**Table:**

Geometry. All e.s.d.'s (except the e.s.d. in the dihedral angle between two l.s. planes) are estimated using the full covariance matrix. The cell e.s.d.'s are taken into account individually in the estimation of e.s.d.'s in distances, angles and torsion angles; correlations between e.s.d.'s in cell parameters are only used when they are defined by crystal symmetry. An approximate (isotropic) treatment of cell e.s.d.'s is used for estimating e.s.d.'s involving l.s. planes.
Refinement. Refinement of *F*^2^ against ALL reflections. The weighted *R*-factor *wR* and goodness of fit *S* are based on *F*^2^, conventional *R*-factors *R* are based on *F*, with *F* set to zero for negative *F*^2^. The threshold expression of *F*^2^ > σ(*F*^2^) is used only for calculating *R*-factors(gt) *etc*. and is not relevant to the choice of reflections for refinement. *R*-factors based on *F*^2^ are statistically about twice as large as those based on *F*, and *R*- factors based on ALL data will be even larger.

### Fractional atomic coordinates and isotropic or equivalent isotropic displacement parameters (Å^2^)

**Table:**

	*x*	*y*	*z*	*U*_iso_*/*U*_eq_	Occ. (<1)
F1	0.6846 (16)	0.5113 (18)	0.0036 (13)	0.121 (4)	0.59 (3)
F1'	0.7330 (17)	0.538 (2)	0.050 (2)	0.105 (5)	0.41 (3)
N1	0.55771 (13)	0.2584 (4)	0.46588 (14)	0.0474 (6)	
N2	0.49151 (15)	0.4615 (4)	0.33021 (15)	0.0608 (7)	
H2	0.4453	0.3666	0.3173	0.073*	
N3	0.61662 (15)	0.0425 (4)	0.59818 (14)	0.0597 (7)	
H3A	0.5670	−0.0413	0.5754	0.072*	
H3B	0.6593	0.0109	0.6523	0.072*	
N4	0.78769 (16)	0.3201 (4)	0.66405 (17)	0.0611 (7)	
O1	0.78379 (14)	0.1726 (4)	0.72145 (14)	0.0792 (7)	
O2	0.86264 (15)	0.4308 (4)	0.68041 (15)	0.0852 (7)	
C1	0.56363 (18)	0.4356 (5)	0.41323 (18)	0.0487 (7)	
C2	0.6413 (2)	0.5883 (5)	0.4429 (2)	0.0604 (8)	
H2A	0.6429	0.7126	0.4061	0.072*	
C3	0.71288 (19)	0.5481 (5)	0.5260 (2)	0.0581 (8)	
H3	0.7649	0.6448	0.5463	0.070*	
C4	0.70977 (17)	0.3639 (4)	0.58174 (18)	0.0488 (7)	
C5	0.62743 (17)	0.2205 (4)	0.54982 (17)	0.0454 (6)	
C6	0.4844 (2)	0.6355 (5)	0.2601 (2)	0.0666 (8)	
H6A	0.5020	0.7765	0.2934	0.080*	
H6B	0.4180	0.6470	0.2188	0.080*	
C7	0.5459 (2)	0.6006 (5)	0.1989 (2)	0.0611 (8)	
C8	0.6128 (14)	0.425 (4)	0.2145 (16)	0.067 (4)	0.59 (3)
H8	0.6195	0.3224	0.2632	0.081*	0.59 (3)
C9	0.669 (3)	0.407 (6)	0.156 (3)	0.097 (7)	0.59 (3)
H9	0.7225	0.3149	0.1712	0.116*	0.59 (3)
C10	0.6390 (18)	0.540 (6)	0.071 (2)	0.087 (6)	0.59 (3)
C11	0.5858 (15)	0.726 (3)	0.0609 (13)	0.082 (4)	0.59 (3)
H11	0.5823	0.8329	0.0145	0.099*	0.59 (3)
C12	0.5373 (18)	0.747 (4)	0.1241 (16)	0.072 (4)	0.59 (3)
H12	0.4958	0.8669	0.1164	0.086*	0.59 (3)
C8'	0.581 (2)	0.398 (6)	0.186 (2)	0.081 (6)	0.41 (3)
H8'	0.5631	0.2733	0.2137	0.097*	0.41 (3)
C9'	0.641 (3)	0.369 (8)	0.134 (3)	0.086 (8)	0.41 (3)
H9'	0.6603	0.2268	0.1225	0.103*	0.41 (3)
C10'	0.673 (3)	0.559 (7)	0.098 (3)	0.078 (7)	0.41 (3)
C11'	0.626 (2)	0.753 (4)	0.099 (2)	0.082 (6)	0.41 (3)
H11'	0.6350	0.8719	0.0630	0.099*	0.41 (3)
C12'	0.566 (2)	0.782 (7)	0.152 (3)	0.080 (7)	0.41 (3)
H12'	0.5393	0.9213	0.1550	0.096*	0.41 (3)

### Atomic displacement parameters (Å^2^)

**Table:**

	*U*^11^	*U*^22^	*U*^33^	*U*^12^	*U*^13^	*U*^23^
F1	0.158 (10)	0.109 (4)	0.135 (8)	0.014 (5)	0.100 (7)	0.009 (5)
F1'	0.124 (10)	0.090 (5)	0.141 (11)	0.018 (6)	0.097 (9)	0.018 (6)
N1	0.0386 (12)	0.0553 (15)	0.0437 (12)	−0.0061 (10)	0.0080 (10)	0.0023 (11)
N2	0.0504 (14)	0.0698 (17)	0.0557 (14)	−0.0039 (11)	0.0096 (12)	0.0163 (12)
N3	0.0514 (13)	0.0679 (17)	0.0481 (12)	−0.0160 (12)	0.0016 (11)	0.0080 (12)
N4	0.0469 (15)	0.0722 (18)	0.0548 (15)	−0.0129 (13)	0.0050 (13)	−0.0155 (14)
O1	0.0631 (13)	0.0991 (18)	0.0574 (12)	−0.0146 (12)	−0.0030 (10)	0.0125 (12)
O2	0.0537 (13)	0.0941 (18)	0.0881 (15)	−0.0231 (12)	−0.0015 (11)	−0.0111 (13)
C1	0.0441 (15)	0.0529 (18)	0.0504 (15)	−0.0001 (13)	0.0180 (13)	0.0025 (14)
C2	0.0638 (18)	0.0505 (19)	0.0666 (19)	−0.0090 (15)	0.0218 (16)	0.0059 (14)
C3	0.0485 (16)	0.0545 (19)	0.0687 (18)	−0.0170 (14)	0.0165 (15)	−0.0079 (16)
C4	0.0418 (15)	0.0527 (18)	0.0478 (15)	−0.0069 (12)	0.0100 (13)	−0.0068 (13)
C5	0.0399 (14)	0.0534 (18)	0.0417 (14)	−0.0054 (13)	0.0121 (12)	−0.0031 (13)
C6	0.0653 (19)	0.071 (2)	0.0636 (18)	0.0142 (16)	0.0224 (16)	0.0190 (16)
C7	0.0670 (19)	0.054 (2)	0.0666 (19)	0.0078 (16)	0.0278 (16)	0.0107 (16)
C8	0.078 (9)	0.056 (7)	0.074 (8)	0.010 (6)	0.034 (7)	0.010 (6)
C9	0.108 (15)	0.074 (14)	0.122 (15)	0.017 (10)	0.056 (12)	0.005 (9)
C10	0.101 (14)	0.081 (11)	0.102 (13)	0.014 (11)	0.063 (10)	0.009 (9)
C11	0.101 (10)	0.073 (7)	0.083 (8)	0.011 (7)	0.044 (7)	0.024 (6)
C12	0.082 (11)	0.064 (9)	0.079 (10)	0.015 (7)	0.039 (8)	0.019 (7)
C8'	0.101 (18)	0.060 (9)	0.090 (16)	−0.004 (11)	0.045 (12)	0.012 (10)
C9'	0.11 (2)	0.057 (12)	0.111 (19)	0.010 (13)	0.069 (16)	0.007 (13)
C10'	0.096 (18)	0.057 (10)	0.11 (2)	−0.003 (13)	0.066 (14)	0.010 (12)
C11'	0.098 (14)	0.067 (9)	0.098 (14)	−0.004 (9)	0.054 (11)	0.019 (10)
C12'	0.091 (17)	0.062 (10)	0.097 (19)	0.003 (11)	0.044 (13)	0.010 (11)

### Geometric parameters (Å, °)

**Table:**

F1—C10	1.39 (3)	C6—H6B	0.9700
F1'—C10'	1.32 (4)	C7—C8'	1.36 (3)
N1—C1	1.341 (3)	C7—C12'	1.38 (4)
N1—C5	1.343 (3)	C7—C12	1.39 (3)
N2—C1	1.341 (3)	C7—C8	1.41 (2)
N2—C6	1.454 (3)	C8—C9	1.40 (4)
N2—H2	0.8600	C8—H8	0.9300
N3—C5	1.327 (3)	C9—C10	1.43 (5)
N3—H3A	0.8600	C9—H9	0.9300
N3—H3B	0.8600	C10—C11	1.35 (4)
N4—O1	1.245 (3)	C11—C12	1.37 (3)
N4—O2	1.245 (3)	C11—H11	0.9300
N4—C4	1.394 (3)	C12—H12	0.9300
C1—C2	1.419 (4)	C8'—C9'	1.38 (6)
C2—C3	1.350 (4)	C8'—H8'	0.9300
C2—H2A	0.9300	C9'—C10'	1.41 (7)
C3—C4	1.392 (4)	C9'—H9'	0.9300
C3—H3	0.9300	C10'—C11'	1.36 (5)
C4—C5	1.436 (3)	C11'—C12'	1.38 (4)
C6—C7	1.506 (4)	C11'—H11'	0.9300
C6—H6A	0.9700	C12'—H12'	0.9300
			
C1—N1—C5	119.7 (2)	C12—C7—C8	118.1 (15)
C1—N2—C6	125.8 (2)	C8'—C7—C6	123.0 (15)
C1—N2—H2	117.1	C12'—C7—C6	118.4 (18)
C6—N2—H2	117.1	C12—C7—C6	119.2 (12)
C5—N3—H3A	120.0	C8—C7—C6	122.6 (10)
C5—N3—H3B	120.0	C9—C8—C7	119 (2)
H3A—N3—H3B	120.0	C9—C8—H8	120.4
O1—N4—O2	119.4 (2)	C7—C8—H8	120.4
O1—N4—C4	121.3 (2)	C8—C9—C10	116 (3)
O2—N4—C4	119.3 (3)	C8—C9—H9	122.0
N2—C1—N1	116.1 (2)	C10—C9—H9	122.0
N2—C1—C2	121.4 (3)	C11—C10—F1	116 (2)
N1—C1—C2	122.5 (2)	C11—C10—C9	123 (3)
C3—C2—C1	118.1 (3)	F1—C10—C9	119 (3)
C3—C2—H2A	120.9	C10—C11—C12	115 (2)
C1—C2—H2A	120.9	C10—C11—H11	122.5
C2—C3—C4	120.9 (3)	C12—C11—H11	122.5
C2—C3—H3	119.6	C11—C12—C7	125 (2)
C4—C3—H3	119.6	C11—C12—H12	117.7
C3—C4—N4	119.2 (2)	C7—C12—H12	117.7
C3—C4—C5	118.3 (2)	C7—C8'—C9'	123 (3)
N4—C4—C5	122.4 (2)	C7—C8'—H8'	118.7
N3—C5—N1	116.4 (2)	C9'—C8'—H8'	118.7
N3—C5—C4	123.2 (2)	C8'—C9'—C10'	119 (4)
N1—C5—C4	120.4 (2)	C8'—C9'—H9'	120.7
N2—C6—C7	115.0 (2)	C10'—C9'—H9'	120.7
N2—C6—H6A	108.5	F1'—C10'—C11'	122 (3)
C7—C6—H6A	108.5	F1'—C10'—C9'	120 (4)
N2—C6—H6B	108.5	C11'—C10'—C9'	116 (4)
C7—C6—H6B	108.5	C10'—C11'—C12'	123 (3)
H6A—C6—H6B	107.5	C10'—C11'—H11'	118.3
C8'—C7—C12'	119 (2)	C12'—C11'—H11'	118.3
C8'—C7—C12	112.9 (17)	C7—C12'—C11'	119 (3)
C12'—C7—C12	21.8 (14)	C7—C12'—H12'	120.7
C8'—C7—C8	22.5 (12)	C11'—C12'—H12'	120.7
C12'—C7—C8	114.4 (19)		
			
C6—N2—C1—N1	−177.6 (2)	C12—C7—C8—C9	1.3 (19)
C6—N2—C1—C2	2.4 (4)	C6—C7—C8—C9	−178.4 (13)
C5—N1—C1—N2	179.7 (2)	C7—C8—C9—C10	−14 (3)
C5—N1—C1—C2	−0.3 (4)	C8—C9—C10—C11	24 (3)
N2—C1—C2—C3	−178.1 (2)	C8—C9—C10—F1	−172.7 (18)
N1—C1—C2—C3	2.0 (4)	F1—C10—C11—C12	176.8 (13)
C1—C2—C3—C4	−0.9 (4)	C9—C10—C11—C12	−19 (3)
C2—C3—C4—N4	176.0 (2)	C10—C11—C12—C7	5(2)
C2—C3—C4—C5	−1.5 (4)	C8'—C7—C12—C11	−21 (2)
O1—N4—C4—C3	171.9 (3)	C12'—C7—C12—C11	89 (8)
O2—N4—C4—C3	−8.0 (4)	C8—C7—C12—C11	3.6 (19)
O1—N4—C4—C5	−10.7 (4)	C6—C7—C12—C11	−176.7 (11)
O2—N4—C4—C5	169.3 (2)	C12'—C7—C8'—C9'	5(3)
C1—N1—C5—N3	179.8 (2)	C12—C7—C8'—C9'	29 (3)
C1—N1—C5—C4	−2.3 (3)	C8—C7—C8'—C9'	−80 (6)
C3—C4—C5—N3	−179.0 (2)	C6—C7—C8'—C9'	−177 (2)
N4—C4—C5—N3	3.6 (4)	C7—C8'—C9'—C10'	5(4)
C3—C4—C5—N1	3.2 (4)	C8'—C9'—C10'—F1'	178 (3)
N4—C4—C5—N1	−174.2 (2)	C8'—C9'—C10'—C11'	−14 (5)
C1—N2—C6—C7	77.2 (3)	F1'—C10'—C11'—C12'	−177.8 (19)
N2—C6—C7—C8'	20.6 (16)	C9'—C10'—C11'—C12'	15 (4)
N2—C6—C7—C12'	−161.0 (15)	C8'—C7—C12'—C11'	−5(2)
N2—C6—C7—C12	174.1 (10)	C12—C7—C12'—C11'	−85 (8)
N2—C6—C7—C8	−6.3 (10)	C8—C7—C12'—C11'	20 (2)
C8'—C7—C8—C9	83 (6)	C6—C7—C12'—C11'	176.8 (13)
C12'—C7—C8—C9	−23 (2)	C10'—C11'—C12'—C7	−6(3)

### Hydrogen-bond geometry (Å, °)

**Table:**

*D*—H···*A*	*D*—H	H···*A*	*D*···*A*	*D*—H···*A*
N3—H3B···O1	0.86	2.03	2.651 (3)	129
N3—H3A···N1^i^	0.86	2.17	3.028 (3)	174
N2—H2···O1^ii^	0.86	2.35	3.060 (3)	141

Symmetry codes: (i) −*x*+1, −*y*, −*z*+1; (ii) *x*−1/2, −*y*+1/2, *z*−1/2.
